# A Computable Phenotype Model for Classification of Men Who Have Sex With Men Within a Large Linked Database of Laboratory, Surveillance, and Administrative Healthcare Records

**DOI:** 10.3389/fdgth.2020.547324

**Published:** 2020-10-06

**Authors:** Travis Salway, Zahid A. Butt, Stanley Wong, Younathan Abdia, Robert Balshaw, Ashleigh J. Rich, Aidan Ablona, Jason Wong, Troy Grennan, Amanda Yu, Maria Alvarez, Carmine Rossi, Mark Gilbert, Mel Krajden, Naveed Z. Janjua

**Affiliations:** ^1^Faculty of Health Sciences, Simon Fraser University, Burnaby, BC, Canada; ^2^British Columbia Centre for Disease Control, Vancouver, BC, Canada; ^3^Centre for Gender and Sexual Health Equity, Vancouver, BC, Canada; ^4^School of Public Health and Health Systems, University of Waterloo, Waterloo, ON, Canada; ^5^George and Fay Yee Centre for Healthcare Innovation, University of Manitoba, Winnipeg, MB, Canada; ^6^School of Population and Public Health, University of British Columbia, Vancouver, BC, Canada; ^7^Department of Medicine, University of British Columbia, Vancouver, BC, Canada; ^8^British Columbia Centre for Disease Control (BCCDC) Public Health Laboratory, Vancouver, BC, Canada; ^9^Department of Pathology and Laboratory Medicine, University of British Columbia, Vancouver, BC, Canada

**Keywords:** sexual and gender minorities, computable phenotypes, big data, HIV, administrative data

## Abstract

**Background:** Most public health datasets do not include sexual orientation measures, thereby limiting the availability of data to monitor health disparities, and evaluate tailored interventions. We therefore developed, validated, and applied a novel computable phenotype model to classify men who have sex with men (MSM) using multiple health datasets from British Columbia, Canada, 1990–2015.

**Methods:** Three case surveillance databases, a public health laboratory database, and five administrative health databases were linked and deidentified (BC Hepatitis Testers Cohort), resulting in a retrospective cohort of 727,091 adult men. Known MSM status from the three disease case surveillance databases was used to develop a multivariable model for classifying MSM in the full cohort. Models were selected using “elastic-net” (GLMNet package) in R, and a final model optimized area under the receiver operating characteristics curve. We compared characteristics of known MSM, classified MSM, and classified heterosexual men.

**Findings:** History of gonorrhea and syphilis diagnoses, HIV tests in the past year, history of visit to an identified gay and bisexual men's clinic, and residence in MSM-dense neighborhoods were all positively associated with being MSM. The selected model had sensitivity of 72%, specificity of 94%. Excluding those with known MSM status, a total of 85,521 men (12% of cohort) were classified as MSM.

**Interpretation:** Computable phenotyping is a promising approach for classification of sexual minorities and investigation of health outcomes in the absence of routinely available self-report data.

## Introduction

Men who have sex with men (MSM) are disproportionately represented in multiple epidemics of public health interest, including HIV, hepatitis C (HCV), hepatitis B (HBV), syphilis, and gonorrhea (1–4). MSM additionally experience numerous mental health and substance use-related inequities, including fourfold greater rates of suicide attempts and twofold greater rates of depression, anxiety, and substance use disorders, as compared with heterosexual men (5–7). These inequities are at least partially attributable to a social stigma attached to minority sexualities, which induces a minority stress response and adaptive behaviors including substance use and sexual risk-taking, in some MSM (8).

In this context, population health databases could be powerful tools for producing new insights into health status and effective disease prevention opportunities for MSM and other sexual and gender minority populations (e.g., sexual minority women, transgender people); however, measurement of sexual and gender minority status is limited in these databases for several reasons. First, sexual orientation and transgender-inclusive measures are not typically recorded in most administrative or laboratory records (3) Second, where collected, MSM status (and analogous minority sexual identities, i.e., gay/lesbian/bisexual) is known to be underreported due to social stigma and related reporting desirability biases. In a recent review, the sensitivity of self-report measures of sexual minority orientation was estimated to be 0.70 (95% credible interval 0.69, 0.71) (specificity: 0.99, 95% credible interval 0.97, 0.99) (6). Third, databases that include MSM or sexual minority self-report status tend to result in small sample sizes which limit the ability to conduct within-group analyses of MSM (9). For these reasons, sexual and gender minority health researchers recommend the use of multiple and novel sampling and measurement strategies for research with MSM and other sexual and gender minorities (9).

“Computable phenotypes” are increasingly being used within electronic health records to identify constructs where direct report or “gold standard” measures are not available, including for social and behavioral health constructs and prediction of HIV-related risk (10–12). While some studies have begun to apply sexual orientation re-classification methods to survey data, models for classifying sexual and gender minority populations within healthcare service databases are under-developed (6). Given the unique healthcare utilization and outcome patterns of MSM—e.g., frequent HIV testing and use of novel biomedical HIV prevention strategies like pre-exposure prophylaxis—linked public health testing and administrative health databases could be used to construct indicators for the identification of MSM (13). Development of MSM computable phenotype models for application within electronic health datasets would enable epidemiologic monitoring of the population-level health status of MSM, evaluation of MSM-focused interventions, and MSM population size estimates.

The British Columbia Hepatitis Testers Cohort (BC-HTC) integrates testing data on HIV and HCV with multiple administrative healthcare databases. With over one million unique individuals, the BC-HTC offers a powerful environment in which to evaluate and implement an MSM computable phenotype. In this report, we present the development, validation, and application of a novel model to classify MSM status using multiple laboratory, administrative healthcare, and public health surveillance datasets from 1990 to 2015, linked and aggregated for the purposes of public health research and monitoring.

## Materials and Methods

### Summary of Methods

**Figure 1:**
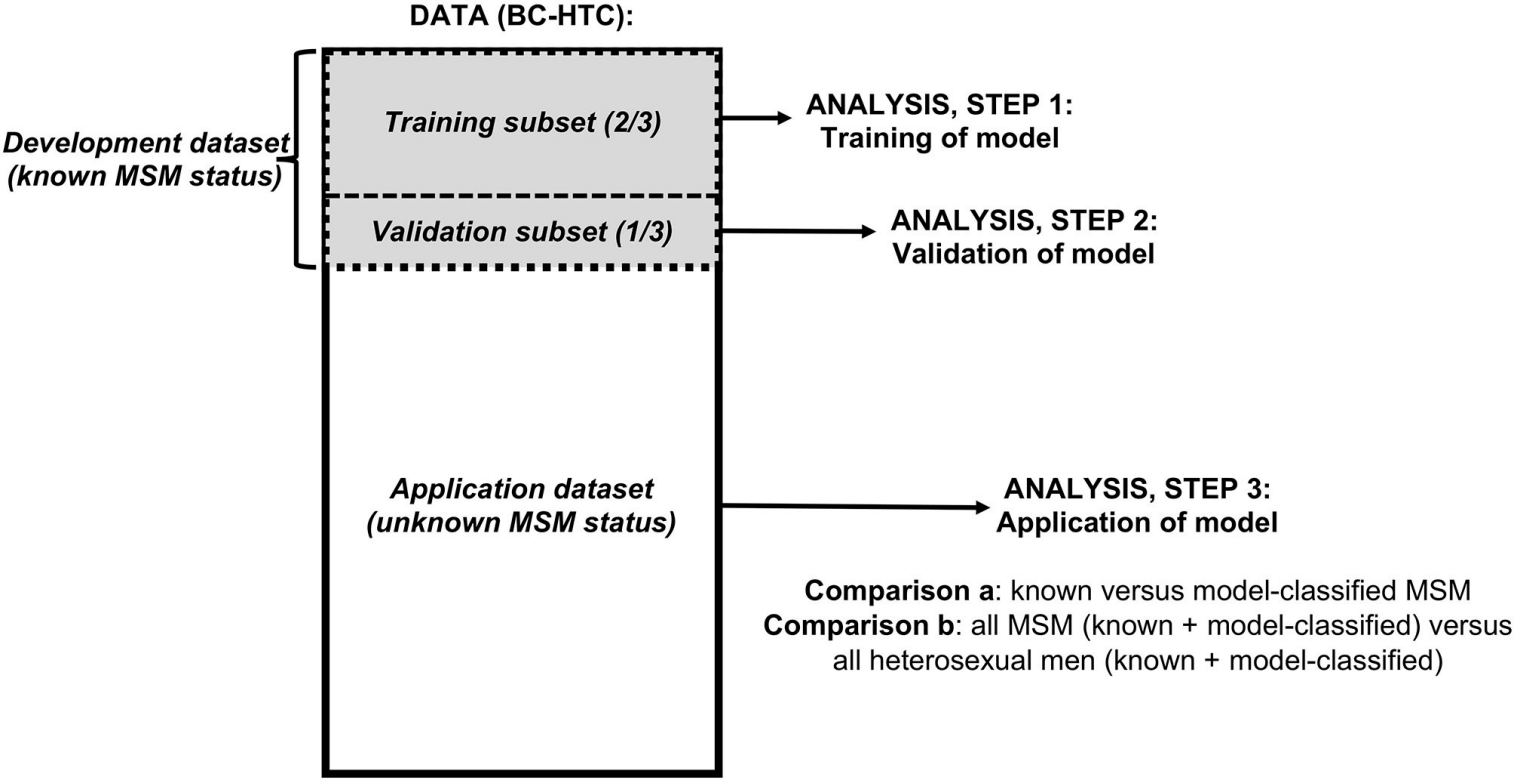
Flowchart of data and analyses used in study of a computable phenotype for men who have sex with men (MSM) within the British Columbia Hepatitis Testers Cohort (BC-HTC).

This study included three steps, corresponding to three subsets of data from the BC-HTC, restricted to men aged 16 years and older (Figure 1). First, we used a subset of data with known MSM status (“develoment dataset”) to train (step 1) and validate (step 2) a computable phenotype model for MSM. The development dataset was randomly divided: 2/3 for model training, 1/3 for validation. We then used our model to classify the remaining records with unknown MSM status within the BC-HTC (“application dataset”) as MSM or heterosexual men (step 3).

### Data

#### Development Dataset

The development dataset comprised three public health case databases: the HIV/AIDS Information System (HAISYS), the Enhanced Hepatitis Strain & Surveillance System (EHSSS), and the Sexually Transmitted Infection Information System (STIIS). A flow diagram documenting the processes for data collection/acquisition and linkages has been published elsewhere (https://ndownloader.figshare.com/files/4824457) (14) HAISYS records surveillance data for new diagnoses of HIV and AIDS in BC, from 1980 onward. At the time of HIV/AIDS diagnosis, detailed demographic and risk factor information—including MSM status—is documented by a provider based on information self-reported by the person diagnosed (risk factor status complete for 74% of cases). EHSSS (2000 onward) and STIIS (1988 onward) also collect risk factors—including MSM status—for new diagnoses of HBV and HCV in EHSSS and syphilis in STIIS, though data completion in these databases is lower (35% in EHSSS, 67% in STIIS). For analysis steps 1 and 2 (development of the model), any man who reported other men as sex partners, including those who reported both men and women as sex partners, was classified as MSM. The recall time-frame for MSM status is not explicitly defined though is generally interpreted by public health nurses as representing partners since last clinic visit, or ever if first clinic visit.

#### Application Dataset

The application dataset included all men in the BC-HTC with unknown MSM status. The BC-HTC includes all individuals (~1.7 million) tested for HCV or HIV at the BC Center for Disease Control Public Health Laboratory (BCCDC-PHL), or reported to public health as a confirmed case of HCV, HBV, or HIV/AIDS, since 1990 (refer to additional publications for more details) (3, 14). The cohort is linked with population-based health databases including those that capture medical visits, hospitalizations, prescription drugs, cancers, and deaths (Supplementary Table 1). More than 95% of HCV and HIV serology, all HIV confirmatory testing, and all HCV RNA testing in BC are performed at the BCCDC-PHL, and thus captured in the BC-HTC.

### Variable Selection

Variables for model building were selected based on theoretical and empirical knowledge about social and health characteristics of MSM (4, 15) and on expert knowledge of the BC-HTC datasets. These variables included: HIV and sexually transmitted infection (STI) testing frequency, previous STI diagnoses, substance use, visit to a clinic providing services to gay and bisexual men (16), prescription for HIV pre-exposure prophylaxis, and residence in area with higher percentage of MSM (Supplementary Table 2). At the time of this study, pre-exposure prophylaxis was recommended by Health Canada for HIV-negative MSM deemed at high risk of acquiring HIV but not publicly funded (17).

### Analysis

#### Step 1: Training the Model

We used the training data (Figure 1) to develop the computable phenotype model. All of the variables described above were included in all models; i.e., the models differed only based on penalization weights, as follows. Many of the explanatory variables were correlated. To select an optimal model accounting for our correlated predictors, we used penalized maximum likelihood—specifically, we used the elastic-net penalty—to fit a logistic regression model. The elastic-net is a weighted combination of the penalties from lasso regression and from ridge regession. The lasso penalty leads to estimated regression coefficients of zero for less important predictors, effectively removing these predictors form the model. The ridge regression penalty encourages sharing of information between correlated predictors. The elastic-net thus leads to parsimonious estimated models with reduced risk of overfitting (18).

In elastic-net there are two tuning parameters, α and λ, that together determine the penalty term: α is a weighting factor from 0 to 1 that determines the balance between lasso regression (α = 1) and ridge regression (α = 0); λ is the parameter controlling the overall strength of the combined penalty. The *glmnet* package can estimate λ to optimize the area under the receiver operator characteristic curve (AUC) but does not provide any support in α tuning. Therefore, we developed models for 11 different values of α (0, 0.1, 0.2, 0.3, 0.4, 0.5, 0.6, 0.7, 0.8, 0.9, 1) so as to maximize AUC, using the validation dataset. We used the *glmnet* package (version 2.0–16) to fit the elastic-net model and also the *caret* package to explore the joint optimization of both α and λ *caret* (version 6.0–84). Both packages were used with the R statistical software (version 3.5.3).

#### Step 2: Validation of Model Perfomance

The performance of the model was assessed by estimating AUC in both the training and the validation subsets of the development data. The difference in AUC between the development and validation subsets, *AUC*_*diff*_, was estimated as a measure of optimism due to overfitting. This process was repeated 1,000 times, and we computed the average of 1,000 *AUC*_*diff*_ (Supplementary Figure 1).

#### Step 3: Application

The selected model was applied to adult men in the entire BC-HTC to estimate the number of MSM in the sample. Two within-sample comparisons were made, quantifying relative differences using prevalence rate ratios with 95% confidence intervals (CI). First, we compared the characteristics of known MSM to model-classified MSM in the application dataset (excluding those with known MSM status). Second, we compared the characteristics of all MSM (known and model-classified) with men who have sex exclusively with women (hereafter, heterosexual men) in the total BC-HTC. Explanatory variables included age, substance use, mental health, STI and blood-borne infection (STBBI) diagnoses, tuberculosis diagnoses, and measures of area-level material and social deprivation (19). Given our large sample size, we have based our intrpetations of the relevance of any differences by their magnitude rather than their *p*-values.

Two sensitivity analyses were conducted to further evaluate the validity of the prediction model. First, we stratified the BC-HTC dataset by those with at least one STI or HIV diagnosis and those with no STI or HIV diagnosis. Second, we stratified the BC-HTC dataset by those with and without an HCV diagnosis. These analyses were performed in order to investigate the potential effects of differential information bias (misclassification) introduced by using set of predictors that relate to particular STI, HIV, and HCV risk factors.

## Results

Table 1 provides the information regarding the sexual behavior of the individuals in the development dataset (i.e., HAISYS, EHSSS, and STIIS databases). The individuals whose status was unknown were excluded from the further analysis. Overall sexual behavior data was available from 25,898 individuals in the development dataset, constituting 66% of all records in these three surveillance databases. 6,280 (24.2%) were identified as MSM, and 19,618 (75.8%) were identified as heterosexual. By database, 20% of EHSSS cases, 63% of HAISYS cases, and 20% of STIIS cases (gonorrhea, chlamydia, and syphilis) were MSM. The charactertics of known MSM and non-MSM in the development dataset are provided in Table 2.

**Table 1:** Proportion of known MSM from different data sources included in development dataset for the development of a computable phenotype model for men who have sex with men within the British Columbia Hepatitis Testers Cohort.

	**EHSSS**		**HAISYS**		**STIIS**		**All 3 databases**	
	**778**		**6,215**		**33,659**		**39,233**	
**Variable**	***N***	**%**	***N***	**%**	***N***	**%**	***N***	**%**
**ALL RECORDS**								
MSM	56	7.2	2,888	46.5	4,502	13.4	6,280	16
HET	218	28	1,710	27.5	17,901	53.2	19,618	50
Unknown	504	64.8	1,617	26	11,256	33.4	13,335	34
**EXCLUDING UNKNOWNS**								
MSM	56	20.4	2,888	62.8	4,502	20.1	6,280	24.2
HET	218	79.6	1,710	37.2	17,901	79.9	19,618	75.8

*EHSSS, Enhanced Hepatitis Strain & Surveillance System; HAISYS, HIV/AIDS Information System; HET, heterosexual men who have sex with women; MSM, men who have sex with men (including men who have sex with men and women); STIIS, Sexually Transmitted Infection Information System, sexually transmitted infection case surveillance database*.

**Table 2:** Characteristics of self-reported men who have sex with men and heterosexual men, HIV, hepatitis, and sexually transmitted infection surveillance case reports, British Columbia, 1980–2015.

	**MSM**		**HET**		**PRR (95% CI)**
	**6,280**		**19,618**		
**Variable**	***N***	**%**	***N***	**%**	
**AGE**					
<15	9	0.1	78	0.4	0.36 (0.18, 0.72)
15–24	1,187	18.9	7,906	40.3	0.47 (0.44, 0.50)
25–34	2,246	35.8	6,801	34.7	1.03 (0.99, 1.07)
35–44	1,616	25.7	2,820	14.4	1.79 (1.70, 1.89)
45–54	865	13.8	1,309	6.7	2.06 (1.90, 2.24)
≥ 55	357	5.7	704	3.6	1.58 (1.40, 1.79)
Illicit drug use	964	15.4	3,233	16.5	0.93 (0.87, 1.00)
Injection drug use	679	10.8	2,053	10.5	1.03 (0.95, 1.12)
Problematic alcohol use	538	8.6	2,084	10.6	0.81 (0.74, 0.88)
Mental illness diagnoses^*^	1,673	26.6	3,069	15.6	1.70 (1.61, 1.79)
HBV	542	8.6	445	2.3	3.80 (3.37, 4.30)
HCV	417	6.6	1,032	5.3	1.26 (1.13, 1.41)
HIV	3,254	51.8	1,740	8.9	5.84 (5.55, 6.15)
STI	4,651	74.1	17,951	91.5	0.81 (0.80, 0.82)
TB	27	0.4	95	0.5	0.89 (0.58, 1.36)
Gonorrhea	2,758	43.9	2,996	15.3	2.87 (2.75, 3.00)
Chlamydia	2,181	34.7	15,689	80	0.43 (0.42, 0.45)
Syphilis	1,654	26.3	740	3.8	6.98 (6.43, 7.58)
**MATERIAL DEPRIVATION**					
Q1	2,647	43.1	4,321	22.3	1.91 (1.84, 1.99)
Q2	1,078	17.6	3,492	18	0.96 (0.91, 1.03)
Q3	756	12.3	3,619	18.7	0.65 (0.61, 0.70)
Q4	803	13.1	3,812	19.7	0.66 (0.61, 0.71)
Q5	853	13.9	4,104	21.2	0.65 (0.61, 0.70)
**SOCIAL DEPRIVATION**					
Q1	488	8	3,107	16.1	0.49 (0.45, 0.54)
Q2	622	10.1	3,271	16.9	0.59 (0.55, 0.64)
Q3	663	10.8	3,379	17.5	0.61 (0.57, 0.66)
Q4	1,158	18.9	4,005	20.7	0.90 (0.85, 0.96)
Q5	3,206	52.2	5,586	28.9	1.79 (1.74, 1.85)

*CI, confidence interval; MSM, men who have sex with men; HET, heterosexual men; PRR, prevalence rate ratio (comparing prevalence in MSM with prevalence in HET)*.

* *defined as at least one hospitalization billing record or two physician (Medical Services Plan) billing records for the following: schizophrenic, bipolar, delusional, nonorganic psychotic, adjustment, anxiety, dissociative, personality, or major depressive disorders. MSM include those who have at least one MSM record at or after age 16*.

### Model Selection

ROC curves from all models overlapped (Supplementary Figure 2), and AUC, sensitivity, and specificity were relatively constant for all values of α (Supplementary Tables 3, 4). Application of these models to the validation dataset showed similar results. We therefore selected the model with α = 0.4 because this model takes advantage of both ridge and lasso models (nearly equal weight) and had higher sensitivity without compromising AUC and specificity. Application of *caret* selected similar values of α and λ and yielded a comparable AUC (0.922) (Supplementary Table 5).

### Relationship of Explanatory Variables With MSM Status

**Figure 2:**
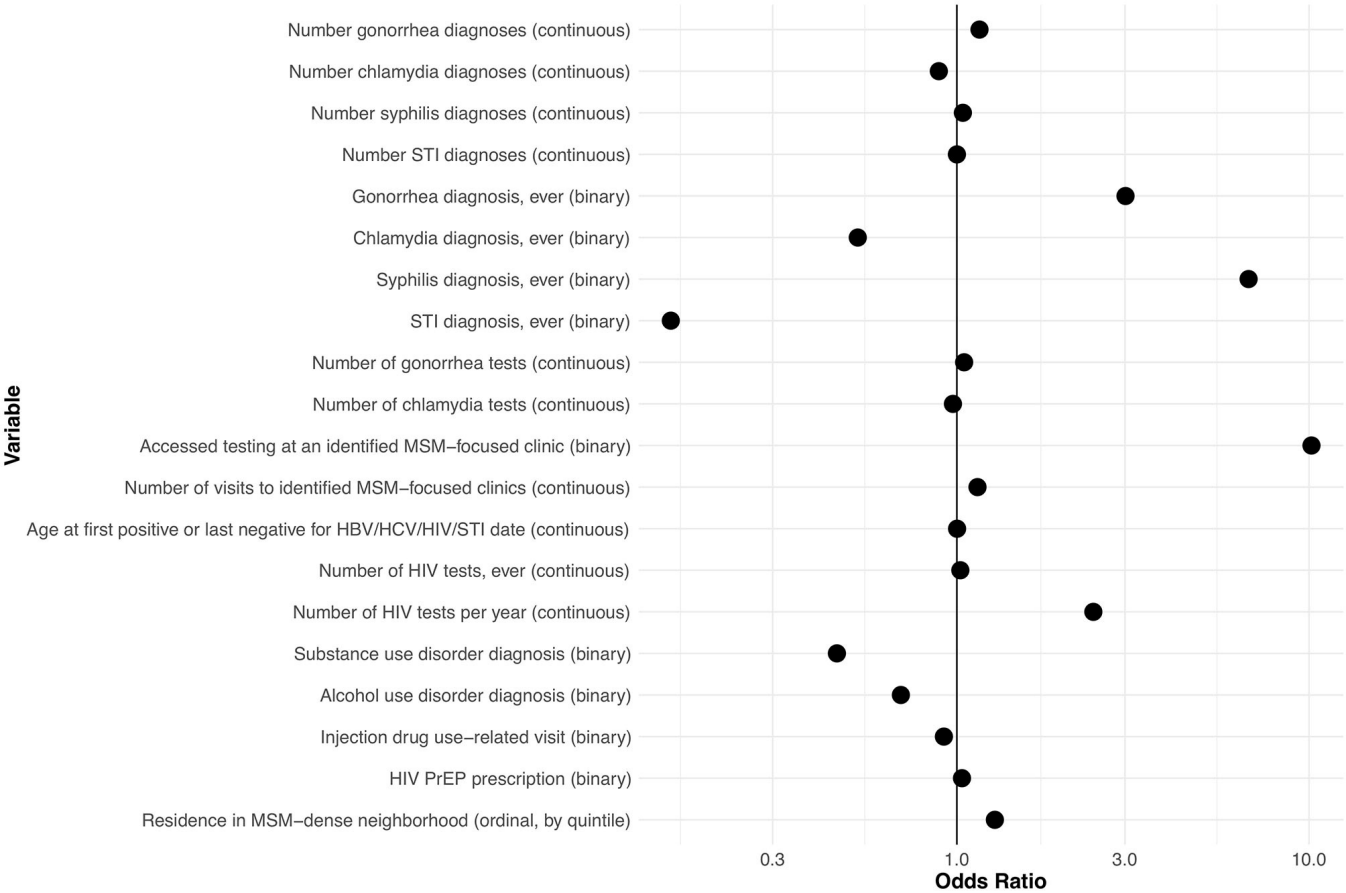
Odds ratios for explanatory variables in a computable phenotype model for men who have sex with men within the British Columbia Hepatitis Testers Cohort. HBV, hepatitis B virus; HCV, hepatitis C virus; MSM, men who have sex with men; PrEP, pre-exposure prophylaxis; STI, sexually transmitted infection (see Supplementary Table 2 for detailed definitions of variables).

History (yes/no) of gonorrhea and syphilis diagnoses, number of HIV tests in the past year, history of visit to an identified gay and bisexual men's clinic, and residence in neighborhoods with higher MSM density were all positively associated with being MSM, with odds ratios > 1.20 (Figure 2). History (yes/no) of chlamydia diagnosis, diagnosis of drug misuse, and diagnosis of alcohol misuse were all inversely associated with being MSM, with odds ratios < 0.83. All other variables had more moderate associations with MSM status (0.83 < OR < 1.20). ORs for variables at other values of α are shown in Supplementary Table 6.

### Characteristics of Classified MSM and Heterosexual Men in BC-HTC

Table 3 provides information regarding the characterisitics of MSM and heterosexual men model-classified within the BC-HTC. A total of 727,091 records were available from adult men in the BC-HTC, 1990–2015, including the 25,898 individuals from the development dataset. Excluding the development dataset, a total of 85,521 MSM (of 701,193 adult men, without sexual behavioral data; 12%) were classified as MSM within the BC-HTC. Model-classified MSM (*N* = 85,521) were older than known MSM (*N* = 6,280). Fewer model-classified MSM had a history of substance use, mental health diagnoses, or history of STBBI, as compared with known MSM. Proportionate distribution of residence by quintiles of material and social deprivation were comparable between model-classified and known MSM.

**Table 3:** Characteristics of known and classified men who have sex with men and heterosexual men in the British Columbia hepatitis testers cohort.

	**MSM, known** ***N*** **=** **6,280**		MSM, classified^*^ ***N*** **=** **85,521**		**PRR^†^ (95% CI)**	**MSM, classified** **+** **known** ***N*** **=** **91,801**		**HET, classified** **+** **known** ***N*** **=** **635,290**		**PRR^‡^ (95% CI)**
**Variable**	**N**	**%**	**N**	**%**		***N***	**%**	***N***	**%**	
**AGE**										
<15	9	0.1	316	0.4	0.39 (0.2, 0.75)	325	0.4	6,156	1	0.37 (0.33, 0.41)
15–24	1,187	18.9	8,983	10.5	1.80 (1.70, 1.90)	10,170	11.1	78,760	12.4	0.89 (0.88, 0.91)
25–34	2,246	35.8	25,544	29.9	1.20 (1.16, 1.24)	27,790	30.3	125,522	19.8	1.53 (1.52, 1.55)
35–44	1,616	25.7	19,496	22.8	1.13 (1.08, 1.18)	21,112	23	123,561	19.4	1.18 (1.17, 1.20)
45–54	865	13.8	13,371	15.6	0.88 (0.83, 0.94)	14,236	15.5	113,330	17.8	0.87 (0.86, 0.88)
≥55	357	5.7	17,811	20.8	0.27 (0.25, 0.30)	18,168	19.8	187,961	29.6	0.67 (0.66, 0.68)
Illicit drug use	964	15.4	2,483	2.9	5.29 (4.93, 5.67)	3,447	3.8	73,811	11.6	0.32 (0.31, 0.33)
Injection drug use	679	10.8	1,625	1.9	5.69 (5.22, 6.20)	2,304	2.5	42,127	6.6	0.38 (0.36, 0.39)
Problematic alcohol use	538	8.6	2,424	2.8	3.02 (2.76, 3.31)	2,962	3.2	65,574	10.3	0.31 (0.30, 0.32)
Mental illness diagnosis	1,673	26.6	12,557	14.7	1.81 (1.74, 1.90)	14,230	15.5	99,639	15.7	0.99 (0.97, 1.00)
HBV	542	8.6	3,941	4.6	1.87 (1.72, 2.04)	4,483	4.9	21,258	3.3	1.46 (1.41, 1.51)
HCV	417	6.6	4,534	5.3	1.25 (1.14, 1.38)	4,951	5.4	41,765	6.6	0.82 (0.80, 0.84)
HIV	3,254	51.8	2,332	2.7	19.00 (18.14, 19.91)	5,586	6.1	4,309	0.7	8.97 (8.63, 9.33)
STI	4,651	74.1	1,617	1.9	39.17 (37.24, 41.20)	6,268	6.8	35,190	5.5	1.23 (1.20, 1.27)
TB	27	0.4	573	0.7	0.64 (0.44, 0.94)	600	0.7	3,457	0.5	1.20 (1.10, 1.31)
Gonorrhea	2,758	43.9	496	0.6	75.72 (69.06, 83.03)	3,254	3.5	6,737	1.1	3.34 (3.21, 3.48)
Chlamydia	2,181	34.7	120	0.1	247.51 (206.33, 296.91)	2,301	2.5	26,239	4.1	0.61 (0.58, 0.63)
Syphilis	1,654	26.3	1,050	1.2	21.45 (19.94, 23.08)	2,704	2.9	3,540	0.6	5.29 (5.03, 5.55)
**MATERIAL DEPRIVATION**										
Q1	2,647	43.1	35,821	43.1	1.01 (0.98, 1.04)	38,468	43.1	121,320	19.3	2.19 (2.17, 2.21)
Q2	1,078	17.6	11,538	13.9	1.27 (1.20, 1.35)	12,616	14.2	124,148	19.8	0.70 (0.69, 0.72)
Q3	756	12.3	9,384	11.3	1.10 (1.02, 1.18)	10,140	11.4	131,131	20.9	0.54 (0.53, 0.55)
Q4	803	13.1	11,173	13.5	0.98 (0.92, 1.05)	11,976	13.4	133,127	21.2	0.62 (0.61, 0.63)
Q5	853	13.9	15,106	18.2	0.77 (0.72, 0.82)	15,959	17.9	117,847	18.8	0.94 (0.92, 0.95)
**SOCIAL DEPRIVATION**										
Q1	488	8	5,911	7.1	1.12 (1.03, 1.23)	6,399	7.2	121,170	19.3	0.37 (0.36, 0.37)
Q2	622	10.1	7,558	9.1	1.12 (1.04, 1.21)	8,180	9.2	118,324	18.9	0.48 (0.47, 0.49)
Q3	663	10.8	8,828	10.6	1.02 (0.95, 1.10)	9,491	10.6	117,662	18.7	0.56 (0.55, 0.57)
Q4	1,158	18.9	17,401	21	0.91 (0.86, 0.96)	18,559	20.8	127,497	20.3	1.01 (0.99, 1.02)
Q5	3,206	52.2	43,324	52.2	1.01 (0.98, 1.03)	46,530	52.2	142,920	22.8	2.25 (2.24, 2.27)

*CI, confidence interval; MSM, men who have sex with men; HET, heterosexual men*;

* *excluding known MSM*;

† *PRR=prevalence rate ratio comparing prevalence in self-reported MSM (development dataset) with prevalence in classified MSM*;

‡ *PRR comparing prevalence in classified + known MSM with prevalence in classified + known HET*.

From Table 3, several differences were discernable while comparing the MSM (both model-classified and known) with heterosexual men (model-classified and known). Proportionately more MSM (42%) than heterosexual men (33%) were <35 years of age. Substance use (including illicit drug use, injection drug use, and alcohol use) was more common among heterosexual men than among MSM. HIV, HBV, gonorrhea, and syphilis, diagnoses were more common among MSM, while HCV diagnoses were more common among heterosexual men. As in the development dataset, more MSM lived in neighborhoods in the lowest quintile of material deprivation (least materially deprived) and those in the highest quintile of social deprivation (most socially deprived), as compared with heterosexual men.

Although the absolute value of the percentages of model-classified MSM and heterosexual men with these characteristics changed with stratification by STBBI history, the relative comparisons between MSM and heterosexual men all remained unchanged (Supplementary Tables 7, 8).

## Discussion

In this study, we applied a computable phenotyping approach to develop a model to classify MSM within a large population-based administrative cohort with over 1 million individuals accessing HIV and HCV testing. This model includes history of STBBI and STBBI testing, use of MSM-tailored clinical services, lack of drug or alcohol misuse diagnoses, and residence in MSM-dense neighborhoods. The selected model had a sensitivity of 72% and specificity of 94% and ultimately classified 12% (*N* = 85,521) of the HIV/HCV testing cohort as MSM.

### Interpretation of Findings

We interpret and evaluate our model with respect to its performance, sub-group comparisons within the BC-HTC, and external validity, in relation to the larger MSM literature. In within-sample comparisons of our model, we found that MSM and heterosexual men were similar between the known-MSM-status datasets and the larger BC-HTC, with a few exceptions. On average, known MSM were older than known heterosexual men, while model-classified MSM were younger than model-classified heterosexual men. This may be partially attributable to the fact that most known MSM came from HIV, gonorrhea, or syphilis case reports—STI diagnoses that tend to occur at older ages (median age among men in BC: 36 years for HIV, 31 years for gonorrhea, 42 years for syphilis)—while most known heterosexual men came from chlamydia case reports, which tend to occur at younger ages (median age among men in BC: 26 years) (20).

Demographic comparisons between model-classified MSM and heterosexual men showed that MSM were younger than heterosexual men, less likely to live in a neighborhood with material deprivation, and more likely to live in a neighborhood with social deprivation. Whether MSM are indeed younger than heterosexual men is difficult to evaluate; theoretical plausibility for this trend comes from generational effects in reduction in the stigma attached to same-gender sexual relations (21), i.e., younger men are more likely to engage in same-gender sexual relationships because they have come of age in a more supportive social environment. However, empirical data comparing the age distributions of sexual minority and heterosexual men have yielded mixed results—likely owing to differences in samples and variables used to identify sexual minorities (i.e., behavioral definitions vs. identity-based definitions of sexual orientation) (15). We may have differentially misclassified older MSM (i.e., with lower sensitivity) because of the predictive variables included in our model. For example, one of the predictors with the largest coefficients was having visited MSM-targeted STBBI testing clinics, some of which are disproportionately accessed by younger men.

The observed patterns of residence in low-material-deprivation and high-social-deprivation neighborhoods can be explained by more carefully examining the composite variables in each of these deprivation indices. The material deprivation index reflects local aggregate measures of education, employment, and income (19). Although most data regarding the socio-economic status of sexual minority men suggests that they experience levels of education, employment, and income that are comparable to or lower than those of heterosexual men (22), the use of certain explanatory variables such as access to MSM-focused clinics, may have biased our MSM predictions to more affluent neighborhoods. The social deprivation index reflects local aggregate measures of marital status and family structure (19). The positive association between predicted MSM status and residence in high-social-deprivation neighborhoods is consistent with other literature that demonstrates that sexual minority men are substantially less likely than heterosexual men to be partnered or married, or to have children (23). Both of these measures, however, rely upon heteronormative assumptions about family and household composition; as noted in other analyses applying area-level measures of social and economic structural factors to sexual minority health, there is a need for the development of sexual minority-specific social and material deprivation measures (24, 25).

Comparative estimates of the burden of STBBI between MSM and heterosexual men, using our computable phenotype model, were consistent with those described elsewhere in the literature on MSM health. In particular, the elevated cumulative prevalence of HIV, gonorrhea, and syphilis in MSM, relative to heterosexual men, are in the same direction as, though smaller in magnitude than, comparative estimates from other studies in North America (2, 26). By contrast, our finding that MSM were no more likely than heterosexual men to experience mental health or substance use disorders is at odds with the multiple systematic reviews that demonstrate a robust 2–4-fold disparity for these outcomes (5–7). The lack of a difference in our dataset between MSM and heterosexual men may be explained by the nature of the population. Our cohort by definition includes individuals at elevated risk of STBBI. Given numerous studies that show associations between STBBI and “syndemic” factors like mental health and substance use disorders (25, 27), it is perhaps not surprising that we failed to detect differences by sexual orientation within this cohort. While most of the above-highlighted findings are consistent with the larger MSM literature, the particular and novel contribution of our study is the development and application of a computable phenotype—combining all of these predictive characteristics—in a way that can be used for in-depth research and public health monitoring within MSM sub-cohorts. We therefore offer these findings to encourage application and exploration of this model, and similar approaches, in comparable public health data-sets in settings beyond BC.

### Limitations

The degree to which this cohort of MSM is representative of MSM in the general population (i.e., including those not accessing HIV/HCV testing) remains to be determined. Future research should employ cross-sample comparisons to better understand which MSM are captured in each of the respective study designs currently employed in MSM and other sexual and gender minority health research. These typically include non-probability venue-based samples, probabilitistic general population samples using self-identification questions, and network-based samples, such as those that employ respondent-driven sampling (28). We note that the number of MSM identified in the BC-HTC (85,521) far exceeds provincial estimates of MSM derived using other methods, e.g., 50,900 in one recent analysis (29). This difference is likely explained by the 94% specificity of our model; i.e., a 6% “false positive” misclassification rate applied to a large population of *N* = 635,290 heterosexual men produces as many as 38,117 heterosexual men who are misclassified as MSM. Future work is needed to improve the specificity of this model, likely through addition of new explanatory variables that may be added as the BC-HTC expands.

We suggest that, as with all observational epidemiologic research, the MSM phenotypic sub-cohort of the BC-HTC will offer some methodologic strengths and some limitations in relation to the other sampling designs. Major strengths of our approach include the large sample size, the ability to integrate multiple data sources, and direct applicability to questions of public health relevance, due to the definitional basis of the cohort including those accessing HIV and hepatitis tests. To optimize classification characteristics, we used elastic-net, implemented with the GLMNet package in R. The elastic-net approach can be particularly beneficial when there are many possibly correlated explanatory variables and there is a concern about the potential for overfitting the regression models.

Drawbacks of our computational phenotype model include misclassification of MSM, a selection bias to disproportionately represent MSM at highest risk of STBBI, and the lack of detailed, self-reported health measures that are typically captured in surveys. Future studies may address these limitations by modeling the effects of misclassification—including those stemming from underreporting of MSM status during STBBI case surveillance (6). Relatedly, MSM status was missing for 34% of all case surveillance records, which may have limited the validity of our model. We further acknowledge that there are other MSM-related explanatory variables that should be explored in future analyses but were unfortunately not available in our dataset. These may include household characteristics (e.g., genders of other household members) and other health characteristics (e.g., receipt of vaccines recommended to sexually active MSM), among others. Future studies should similarly explore more parsimonious models that reduce the number of explanatory variables, including models that may sacrifice sensitivity to achieve high specificity.

Finally, we acknowledge the critical importance of ensuring that patient privacy is protected in all stages of computable phenotyping—particularly for a topic that remains stigmatized in our society (i.e., sexual minority status). As others have asserted (10, 30), technological advances associated with big data that enable new analyses for public good need not (and must not) compromise principles of confidentiality. The BC-HTC uses several measures to ensure protection of sensitive data, including: (a) de-identification of linked data used for analysis; (b) storing all data within a robust security system (14); and (c) ensuring there are no ways for newly derived variables (e.g., model-classified MSM status) to return to patient charts or other electronic databases where individual characteristics could be read or misused. We further suggest that there is a need for ongoing research and monitoring to understand sexual minority community perspectives on the use of computable phenotype tools—both to understand how these methods can be applied to urgent questions of community interest and to address any real or perceived threats to individual privacy, community representations, and other ethical concerns yet to be established.

## Conclusions

Computable phenotyping is a promising approach for the identification of sexual and gender minorities, in order to strengthen efforts in population health monitoring, particularly in the absence of routinely available self-report data. Our computable phenotype model had classification characteristics similar to interviewer-elicited survey measures. This method may ultimately allow for larger samples and triangulation between other sexual minority samples, with their own particular limitations (9). In this context, we recommend greater application and exploration of computable phenotyping in sexual minority health research.

## Data Availability Statement

The data analyzed in this study is subject to the following licenses/restrictions: Data are sensitive in nature and stewarded by appropriate public health authorities in British Columbia. Requests to access these datasets should be directed to naveed.janjua@bccdc.ca.

## Ethics Statement

This study was reviewed and approved by the University of British Columbia Research Ethics Board (H14-01649). Written informed consent for participation was not required for this study in accordance with the national legislation and the institutional requirements.

## Author Contributions

ZB, RB, and NJ: study design. ZB, SW, JW, AY, MA, CR, MG, MK, and NJ: collection and construction of data cohort. TS, SW, and YA: analysis. TS and ZB: preparation of manuscript. TS, ZB, SW, YA, RB, and NJ: tables and figures. AR, AA, JW, TG, MG, and NJ: contributions to literature review, interpretation, and discussion of model results. All authors contributing to drafting this manuscript and have approved it for submission.

## Conflict of Interest

TG was an investigator on research grants funded by Gilead and Merck. MK has received grant funding via his institution from Roche Molecular Systems, Boehringer Ingelheim, Merck, Siemens Healthcare Diagnostics, and Hologic Inc. The remaining authors declare that the research was conducted in the absence of any commercial or financial relationships that could be construed as a potential conflict of interest.
