# Supplementary material for: A Computable Phenotype Model for Classification of Men Who Have Sex With Men Within a Large Linked Database of Laboratory, Surveillance, and Administrative Healthcare Records
Source: Front Digit Health. 2020 Oct 6;2:547324. doi: 10.3389/fdgth.2020.547324 (PMC8521949; doi:10.3389/fdgth.2020.547324)
Supplement: Supplementary file 1 [file Data_Sheet_1.docx]

**Supplementary Materials**

**Supplementary Table 1.** Criteria and data sources for the BC Hepatitis Testers Cohort (BC-HTC)

**Criteria and Data Sources for the BC Hepatitis Testers Cohort (BC-HTC)
Web:** [**https://bchtc.med.ubc.ca/**](https://bchtc.med.ubc.ca/)

| **Criteria for Inclusion in BC-HTC** | |
| --- | --- |
|  | |
| All individuals:   - tested at the BCCDC Public Health Laboratory (BC-PHL ) for HCV or HIV OR - reported to BC public health as a confirmed case of HCV, HIV or AIDS, HBV, or active TB OR - included in BC Enhanced Strain Surveillance System (EHSSS) as an acute HBV or HCV case | |
| All individuals meeting at least one the above criteria were linked internally across all their tests and case reports. Those with a valid personal health number (PHN) were then sent for deterministic linkage with the province-wide Cancer and Ministry of Health (MoH) datasets. | |
| **Provincial Communicable Disease Data Sources:** | **Data Date Ranges:** |
| BC-PHMRL HIV laboratory testing datasets (tests: ELISA, Western blot, NAAT, p24, culture) | 1988–2015 |
| BC-PHMRL HCV laboratory tests datasets (tests: antibody, HCV RNA, genotyping) | 1992–2019, Oct |
| HIV/AIDS Information System (HAISYS) (public health HIV/AIDS case reports) | 1980–2015 |
| Integrated Public Health information System (iPHIS) (public health case reports of HCV, HBV, and TB) | 1990–2015 |
| Enhanced Strain Surveillance System (EHSSS) (risk factor data on a subset of acute HCV and acute HBV cases) | 2000–2013 |
| **Cancer and MoH Administrative Data Sources:** | **Data Date Ranges:** |
| Client Roster (CR) (Registry of enrollment in the universal public health insurance plan including residential history) | 1990–2016 |
| BC Cancer Registry (BCCR) (primary tumour registry, excludes metastatic cancers) | 1923–2016 |
| Discharge Abstracts Dataset (DAD) (hospitalization records)^S1^ | 1985–2015 |
| Medical Services Plan (MSP) (physician diagnostic and billing data)^S2^ | 1990–2015 |
| PharmaCare/PharmaNet (Pharma) (prescription drug dispensations)^S3, S4^ | 1985–2019, Jun |
| BC Vital Statistics (VS) (deaths registry)^S5^ | 1985–2019, Jun |
| NACRS (Emergency Departments) | 2012–2015 |
| Ethnicity (based on name recognition) | 1980–2015 |
| Chronic Disease Registry^S6^ | 1992–2015 |
| The final BC-HTC comprises all individuals successfully linked on PHN to the MoH Client Roster^S7^ (a registry of all BC residents enrolled in the publicly-funded universal healthcare system) | |

HCV: Hepatitis C Virus; HBV: Hepatitis B Virus; HIV/AIDS: Human Immunodeficiency Virus/Acquired Immunodeficiency Syndrome; BC-PHMRL: BC Public Health Microbiology and Reference Laboratory: RNA: Ribonucleic Acid; PCR: Polymerase Chain Reaction.

**Supplementary References**:

1. British Columbia Ministry of Health [creator]. Discharge Abstract Database (Hospital Separations). British Columbia Ministry of Health [publisher]. Data Extract. MOH (2013). 2016. <https://www2.gov.bc.ca/gov/content/health/conducting-health-research-evaluation/data-access-health-data-central/health-authorities>
2. British Columbia Ministry of Health [creator]. Medical Services Plan (MSP) Payment Information File. British Columbia Ministry of Health [publisher]. Data Extract. MOH (2013). 2016. <https://www2.gov.bc.ca/gov/content/health/conducting-health-research-evaluation/data-access-health-data-central/health-authorities>
3. British Columbia Ministry of Health [creator]. PharmaCare. British Columbia Ministry of Health [publisher]. Data Extract. MOH (2013). 2019. <https://www2.gov.bc.ca/gov/content/health/conducting-health-research-evaluation/data-access-health-data-central/health-authorities>
4. British Columbia Ministry of Health [creator]. PharmaNet. British Columbia Ministry of Health [publisher]. Data Extract. MOH (2013). 2019. <https://www2.gov.bc.ca/gov/content/health/conducting-health-research-evaluation/data-access-health-data-central/health-authorities>
5. BC Vital Statistics Agency [creator]. Vital Statistics Deaths. BC Vital Statistics Agency [publisher]. Data Extract. BC Vital Statistics Agency (2014). 2019. <https://www2.gov.bc.ca/gov/content/health/conducting-health-research-evaluation/data-access-health-data-central/health-authorities>
6. British Columbia Ministry of Health [creator]. Chronic Disease Registry. British Columbia Ministry of Health [publisher]. Data Extract. MOH. (2016) 2016. <https://www2.gov.bc.ca/gov/content/health/conducting-health-research-evaluation/data-access-health-data-central/health-authorities>
7. British Columbia Ministry of Health [creator]. Client Roster (Client Registry System/Enterprise Master Patient Index). British Columbia Ministry of Health [publisher]. Data Extract. MOH (2013). 2016. <https://www2.gov.bc.ca/gov/content/health/conducting-health-research-evaluation/data-access-health-data-central/health-authorities>

**Supplementary Table 2.** Description of variables

| **Description** | **Data source(s)** |
| --- | --- |
| Number of gonorrhea cases | DAD, MSP, STIIS |
| Number of chlamydia cases | DAD, MSP, STIIS |
| Number of syphilis cases | DAD, MSP, STIIS |
| Number of STI cases (gonorrhea, chlamydia, or syphilis) | DAD, MSP, STIIS |
| Gonorrhea flag (yes/no) | DAD, MSP, STIIS |
| Chlamydia flag (yes/no) | DAD, MSP, STIIS |
| Syphilis flag (yes/no) | DAD, MSP, STIIS |
| STI flag (yes/no; gonorrhea, chlamydia, or syphilis) | DAD, MSP, STIIS |
| Number of gonorrhea tests based on MSP (fee item codes) | MSP |
| Number of chlamydia tests based on MSP (fee item codes) | MSP |
| MSM clinic visited flag (yes/no) | HAISYS, STIIS, Laboratory information systems |
| Number of MSM clinic visits | HAISYS, STIIS, Laboratory information systems |
| Age at first positive or last negative for HBV/HCV/HIV/STI date | DAD, MSP, STIIS |
| Number of HIV tests | Laboratory information systems |
| Number of HIV tests per year | Laboratory information systems |
| Drug misused (before Dec 31, 2015) | DAD (at least one record), MSP (at least two records) |
| Alcohol misused (before Dec 31, 2015) | DAD (at least one record), MSP (at least two records) |
| Injection drug use (before Dec 31, 2015) | DAD (at least one record), MSP (at least two records) |
| HIV PrEP flag (yes/no) | Pharmanet |
| MSM FSA quintile | HAISYS, STIIS, EHSSS |

**Note.** DAD=Discharge Abstract Database, hospitalization records; EHSSS=Enhanced Hepatitis Strain & Surveillance System; HAISYS=HIV/AIDS Information System; MSP=Medical Services Plan, physician billing records; PrEP=pre-exposure prophylaxis; STIIS=STI Information System, sexually transmitted infection (STI) case surveillance database;

**Supplementary Figure 1.** Methods for calculating adjusted AUC

**Supplementary Figure 2**. ROC curves for various alphas
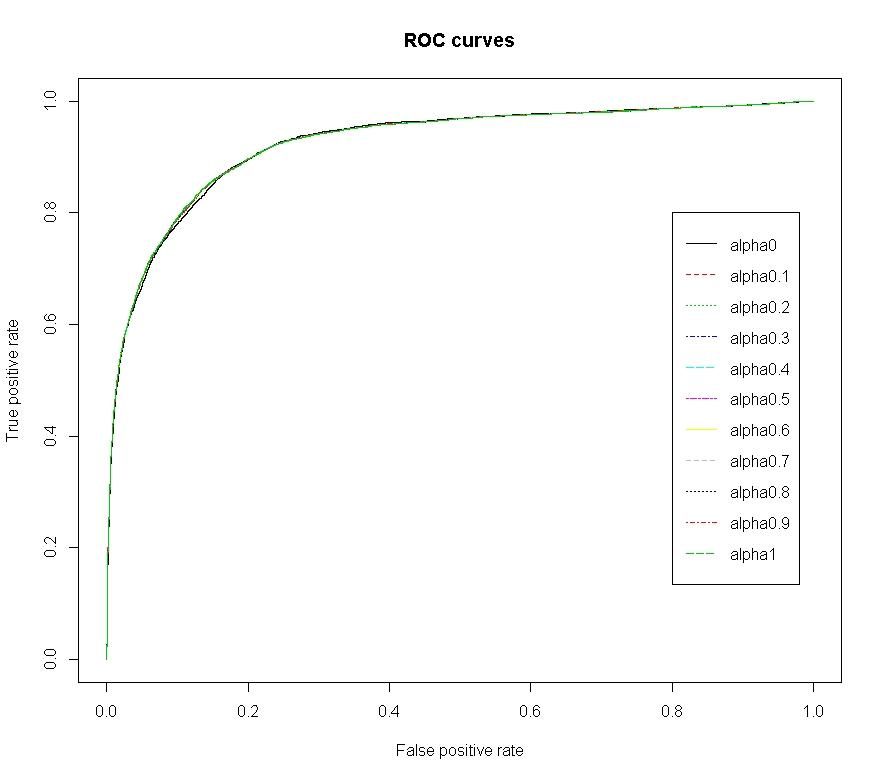


**Supplementary Table 3**. Accuracy, sensitivity, specificity, and AUC using the complete development dataset

| Alpha | lambda.min_all_ | Accuracy_all_ | Sensitivity_all_ | Specificity_all_ | AUC_all_ |
| --- | --- | --- | --- | --- | --- |
| 0 | 0.0288 | 0.8829 | 0.7051 | 0.9398 | 0.9243 |
| 0.1 | 0.0039 | 0.8840 | 0.7202 | 0.9364 | 0.9248 |
| 0.2 | 0.0023 | 0.8844 | 0.7218 | 0.9364 | 0.9248 |
| 0.3 | 0.0008 | 0.8842 | 0.7232 | 0.9357 | 0.9248 |
| 0.4 | 0.0010 | 0.8842 | 0.7225 | 0.9360 | 0.9248 |
| 0.5 | 0.0009 | 0.8842 | 0.7223 | 0.9360 | 0.9248 |
| 0.6 | 0.0005 | 0.8840 | 0.7228 | 0.9357 | 0.9248 |
| 0.7 | 0.0005 | 0.8842 | 0.7228 | 0.9358 | 0.9248 |
| 0.8 | 0.0004 | 0.8841 | 0.7228 | 0.9358 | 0.9248 |
| 0.9 | 0.0004 | 0.8842 | 0.7228 | 0.9359 | 0.9248 |
| 1 | 0.0004 | 0.8842 | 0.7228 | 0.9358 | 0.9248 |

**Supplementary Table 4.** Accuracy, sensitivity, specificity, and AUC using the training and validation datasets

| Alpha | lambda.min_train_ | Accuracy_train_ | Sensitivity_train_ | Specificity_train_ | AUC_train_ | Accuracy_validate_ | Sensitivity_validate_ | Specificity_validate_ | AUC_validate_ |
| --- | --- | --- | --- | --- | --- | --- | --- | --- | --- |
| 0 | 0.0288 | 0.8660 | 0.8375 | 0.8445 | 0.9240 | 0.866 | 0.8372 | 0.8442 | 0.9235 |
| 0.1 | 0.0030 | 0.8511 | 0.8557 | 0.8546 | 0.9247 | 0.8517 | 0.8545 | 0.8538 | 0.9240 |
| 0.2 | 0.0020 | 0.8500 | 0.8570 | 0.8553 | 0.9247 | 0.8506 | 0.8556 | 0.8544 | 0.9239 |
| 0.3 | 0.0016 | 0.8493 | 0.8576 | 0.8556 | 0.9247 | 0.8502 | 0.8561 | 0.8546 | 0.9239 |
| 0.4 | 0.0013 | 0.8490 | 0.8580 | 0.8558 | 0.9247 | 0.8496 | 0.8567 | 0.8550 | 0.9239 |
| 0.5 | 0.0011 | 0.8488 | 0.8582 | 0.8560 | 0.9247 | 0.8492 | 0.8571 | 0.8551 | 0.9239 |
| 0.6 | 0.0009 | 0.8485 | 0.8586 | 0.8562 | 0.9246 | 0.8493 | 0.8570 | 0.8551 | 0.9239 |
| 0.7 | 0.0008 | 0.8483 | 0.8588 | 0.8562 | 0.9246 | 0.8492 | 0.8571 | 0.8552 | 0.9239 |
| 0.8 | 0.0007 | 0.8483 | 0.8588 | 0.8562 | 0.9246 | 0.8489 | 0.8574 | 0.8554 | 0.9239 |
| 0.9 | 0.0007 | 0.8483 | 0.8588 | 0.8563 | 0.9246 | 0.8489 | 0.8574 | 0.8554 | 0.9239 |
| 1 | 0.0006 | 0.8481 | 0.8590 | 0.8564 | 0.9246 | 0.8490 | 0.8574 | 0.8553 | 0.9239 |

**Supplementary Table 5.** Accuracy, sensitivity, specificity, and AUC using the training and testing dataset using the CARET package

| Alpha | lambda.min_train_ | Accuracy_train_ | Sensitivity_train_ | Specificity_train_ | AUC_train_ | Accuracy_validate_ | Sensitivity_validate_ | Specificity_validate_ | AUC_validate_ |
| --- | --- | --- | --- | --- | --- | --- | --- | --- | --- |
| 0.4951 | 0.0390 | 0.8816 | 0.6912 | 0.9426 | 0.9235 | 0.8809 | 0.6898 | 0.9420 | 0.9228 |

**Supplementary Table 6**. Odds Ratios for Explanatory Variables in a Computable Phenotype Model for Men Who Have Sex With Men Within the British Columbia Hepatitis Testers Cohort, at varying values of alpha.

|  | **Alpha** | | | | | | | | | | |
| --- | --- | --- | --- | --- | --- | --- | --- | --- | --- | --- | --- |
| **Variable** | **0.0** | **0.1** | **0.2** | **0.3** | **0.4** | **0.5** | **0.6** | **0.7** | **0.8** | **0.9** | **1.0** |
| (Intercept) | 0.1866 | 0.2415 | 0.2497 | 0.2577 | 0.2574 | 0.2583 | 0.2599 | 0.2603 | 0.2607 | 0.2609 | 0.2612 |
| Number gonorrhea diagnoses (continuous) | 1.2029 | 1.1803 | 1.1709 | 1.1611 | 1.1592 | 1.1567 | 1.1554 | 1.1542 | 1.1533 | 1.1522 | 1.1516 |
| Number chlamydia diagnoses (continuous) | 0.8318 | 0.8804 | 0.8877 | 0.8870 | 0.8885 | 0.8893 | 0.8880 | 0.8884 | 0.8883 | 0.8888 | 0.8888 |
| Number syphilis diagnoses (continuous) | 1.0852 | 1.0500 | 1.0461 | 1.0409 | 1.0400 | 1.0389 | 1.0383 | 1.0378 | 1.0374 | 1.0370 | 1.0367 |
| Number STI diagnoses (continuous) | 1.0288 | 1.0036 | 1 | 1 | 1 | 1 | 1 | 1 | 1 | 1 | 1 |
| Gonorrhea diagnosis, ever (binary) | 1.8362 | 2.6415 | 2.8126 | 3.0356 | 3.0118 | 3.0343 | 3.0906 | 3.0968 | 3.1093 | 3.1117 | 3.1204 |
| Chlamydia diagnosis, ever (binary) | 0.4914 | 0.4994 | 0.5085 | 0.5267 | 0.5232 | 0.5243 | 0.5302 | 0.5302 | 0.5312 | 0.5307 | 0.5314 |
| Syphilis diagnosis, ever (binary) | 3.2131 | 5.7372 | 6.2073 | 6.7913 | 6.7321 | 6.7894 | 6.9301 | 6.9467 | 6.9778 | 6.9838 | 7.0055 |
| STI diagnosis, ever (binary) | 0.3266 | 0.1827 | 0.1684 | 0.1522 | 0.1541 | 0.1529 | 0.1492 | 0.1489 | 0.1482 | 0.1482 | 0.1477 |
| Number of gonorrhea tests (continuous) | 1.0218 | 1.0406 | 1.0422 | 1.0540 | 1.0487 | 1.0480 | 1.0526 | 1.0516 | 1.0519 | 1.0507 | 1.0509 |
| Number of chlamydia tests (continuous) | 0.9861 | 0.9786 | 0.9784 | 0.9706 | 0.9745 | 0.9751 | 0.9719 | 0.9726 | 0.9725 | 0.9734 | 0.9733 |
| Accessed testing at an identified MSM-focused clinic (binary) | 6.8728 | 9.8078 | 10.0333 | 10.1382 | 10.1594 | 10.1767 | 10.1722 | 10.1814 | 10.1845 | 10.1936 | 10.1956 |
| Number of visits to identified MSM-focused clinics (continuous) | 1.0897 | 1.1275 | 1.1345 | 1.1468 | 1.1443 | 1.1449 | 1.1485 | 1.1485 | 1.1491 | 1.1489 | 1.1493 |
| Age at first positive or last negative for HBV/HCV/HIV/STI date (continuous) | 1.0051 | 1.0018 | 1.0014 | 1.0012 | 1.0011 | 1.0011 | 1.0011 | 1.0010 | 1.0010 | 1.0010 | 1.0010 |
| Number of HIV tests, ever (continuous) | 1.0382 | 1.0265 | 1.0244 | 1.0229 | 1.0226 | 1.0223 | 1.0222 | 1.0220 | 1.0219 | 1.0218 | 1.0217 |
| Number of HIV tests per year (continuous) | 2.0156 | 2.3488 | 2.3965 | 2.4406 | 2.4408 | 2.4466 | 2.4539 | 2.4567 | 2.4591 | 2.4613 | 2.4630 |
| Substance use disorder diagnosis (binary) | 0.6364 | 0.4917 | 0.4743 | 0.4543 | 0.4566 | 0.4550 | 0.4505 | 0.4500 | 0.4491 | 0.4490 | 0.4483 |
| Alcohol use disorder diagnosis (binary) | 0.7213 | 0.6959 | 0.6960 | 0.6913 | 0.6938 | 0.6943 | 0.6924 | 0.6929 | 0.6929 | 0.6935 | 0.6934 |
| Injection drug use-related visit (binary) | 0.8046 | 0.8789 | 0.9002 | 0.9164 | 0.9191 | 0.9227 | 0.9247 | 0.9266 | 0.9278 | 0.9295 | 0.9303 |
| HIV PrEP prescription (binary) | 1.1523 | 1.0483 | 1.0353 | 1.0425 | 1.0334 | 1.0302 | 1.0355 | 1.0332 | 1.0328 | 1.0304 | 1.0301 |
| Residence in MSM-dense neighborhood (ordinal, by quintile) | 1.2466 | 1.2772 | 1.2794 | 1.2836 | 1.2824 | 1.2824 | 1.2837 | 1.2835 | 1.2836 | 1.2834 | 1.2835 |

**Note.** HBV=hepatitis B virus; HCV=hepatitis C virus; MSM=men who have sex with men; PrEP= pre-exposure prophylaxis; STI=sexually transmitted infection. See Supplementary Table 2 for detailed definitions of variables.

**Supplementary Table 7**. Characteristics of model-classified MSM and heterosexual men by HIV/STI status (at least one record at or after age 16)

|  | **MSM**  **with STI/HIV** | | **HET**  **with STI/HIV** | | **MSM**  **without STI/HIV** | | **HET**  **without STI/HIV** | |
| --- | --- | --- | --- | --- | --- | --- | --- | --- |
|  | **N=3,697** | | **N=19,678** | | **N=81,824** | | **N=595,994** | |
| **Variable** | **N** | **%** | **N** | **%** | **N** | **%** | **N** | **%** |
| Age |  |  |  |  |  |  |  |  |
| < 15 | 97 | 2.6 | 768 | 3.9 | 219 | 0.3 | 5,310 | 0.9 |
| 15 - 24 | 280 | 7.6 | 5,989 | 30.4 | 8,703 | 10.6 | 64,865 | 10.9 |
| 25 - 34 | 1,040 | 28.1 | 6,038 | 30.7 | 24,504 | 29.9 | 112,683 | 18.9 |
| 35 - 44 | 1,135 | 30.7 | 3,280 | 16.7 | 18,361 | 22.4 | 117,461 | 19.7 |
| 45 - 54 | 680 | 18.4 | 1,833 | 9.3 | 12,691 | 15.5 | 110,188 | 18.5 |
| ≥ 55 | 465 | 12.6 | 1,770 | 9 | 17,346 | 21.2 | 185,487 | 31.1 |
| Illicit drug use | 550 | 14.9 | 4,264 | 21.7 | 1,933 | 2.4 | 66,314 | 11.1 |
| Injection drug use | 417 | 11.3 | 2,973 | 15.1 | 1,208 | 1.5 | 37,101 | 6.2 |
| Problematic alcohol use | 357 | 9.7 | 2,990 | 15.2 | 2,067 | 2.5 | 60,500 | 10.2 |
| Mental illness diagnosis | 1,133 | 30.6 | 3,981 | 20.2 | 11,424 | 14 | 92,589 | 15.5 |
| HBV | 415 | 11.2 | 782 | 4 | 3,526 | 4.3 | 20,031 | 3.4 |
| HCV | 650 | 17.6 | 2,192 | 11.1 | 3,884 | 4.7 | 38,541 | 6.5 |
| HIV | 2,332 | 63.1 | 2,569 | 13.1 | 0 | 0 | 0 | 0 |
| STI | 1,617 | 43.7 | 17,239 | 87.6 | 0 | 0 | 0 | 0 |
| TB | 51 | 1.4 | 161 | 0.8 | 522 | 0.6 | 3,201 | 0.5 |
| Gonorrhea | 496 | 13.4 | 3,741 | 19 | 0 | 0 | 0 | 0 |
| Chlamydia | 120 | 3.2 | 10,550 | 53.6 | 0 | 0 | 0 | 0 |
| Syphilis | 1,050 | 28.4 | 2,800 | 14.2 | 0 | 0 | 0 | 0 |
| Material Deprivation |  |  |  |  |  |  |  |  |
| Q1 | 1,394 | 38.6 | 3,674 | 19.1 | 34,427 | 43.4 | 113,325 | 19.2 |
| Q2 | 611 | 16.9 | 3,457 | 18 | 10,927 | 13.8 | 117,199 | 19.9 |
| Q3 | 402 | 11.1 | 3,553 | 18.5 | 8,982 | 11.3 | 123,959 | 21 |
| Q4 | 454 | 12.6 | 4,048 | 21.1 | 10,719 | 13.5 | 125,267 | 21.3 |
| Q5 | 753 | 20.8 | 4,469 | 23.3 | 14,353 | 18.1 | 109,274 | 18.6 |
| Social Deprivation |  |  |  |  |  |  |  |  |
| Q1 | 228 | 6.3 | 2,769 | 14.4 | 5,683 | 7.2 | 115,294 | 19.6 |
| Q2 | 302 | 8.4 | 3,263 | 17 | 7,256 | 9.1 | 111,790 | 19 |
| Q3 | 362 | 10 | 3,477 | 18.1 | 8,466 | 10.7 | 110,806 | 18.8 |
| Q4 | 670 | 18.5 | 4,073 | 21.2 | 16,731 | 21.1 | 119,419 | 20.3 |
| Q5 | 2,052 | 56.8 | 5,619 | 29.3 | 41,272 | 52 | 131,715 | 22.4 |

**Supplementary Table 8**. Characteristics of model-classified MSM and heterosexual men by HCV status (at least one record at or after age 16)

|  | **MSM with HCV** | | **HET**  **with HCV** | | **MSM**  **without HCV** | | **HET**  **without HCV** | |
| --- | --- | --- | --- | --- | --- | --- | --- | --- |
|  | **N=4,534** | | **N=40,733** | | **N=80,987** | | **N=574,939** | |
| **Variable** | **N** | **%** | **N** | **%** | **N** | **%** | **N** | **%** |
| Age |  |  |  |  |  |  |  |  |
| < 15 | 19 | 0.4 | 148 | 0.4 | 297 | 0.4 | 5,930 | 1 |
| 15 - 24 | 292 | 6.4 | 1,823 | 4.5 | 8,691 | 10.7 | 69,031 | 12 |
| 25 - 34 | 1,069 | 23.6 | 7,971 | 19.6 | 24,475 | 30.2 | 110,750 | 19.3 |
| 35 - 44 | 1,416 | 31.2 | 12,890 | 31.6 | 18,080 | 22.3 | 107,851 | 18.8 |
| 45 - 54 | 1,088 | 24 | 11,150 | 27.4 | 12,283 | 15.2 | 100,871 | 17.5 |
| ≥ 55 | 650 | 14.3 | 6,751 | 16.6 | 17,161 | 21.2 | 180,506 | 31.4 |
| Illicit drug use | 1,125 | 24.8 | 20,224 | 49.7 | 1,358 | 1.7 | 50,354 | 8.8 |
| Injection drug use | 854 | 18.8 | 13,924 | 34.2 | 771 | 1 | 26,150 | 4.5 |
| Problematic alcohol use | 654 | 14.4 | 13,524 | 33.2 | 1,770 | 2.2 | 49,966 | 8.7 |
| Mental illness diagnosis | 1,223 | 27 | 11,792 | 28.9 | 11,334 | 14 | 84,778 | 14.7 |
| HBV | 405 | 8.9 | 3,024 | 7.4 | 3,536 | 4.4 | 17,789 | 3.1 |
| HCV | 4,534 | 100 | 40,733 | 100 | 0 | 0 | 0 | 0 |
| HIV | 579 | 12.8 | 1,622 | 4 | 1,753 | 2.2 | 947 | 0.2 |
| STI | 127 | 2.8 | 616 | 1.5 | 1,490 | 1.8 | 16,623 | 2.9 |
| TB | 44 | 1 | 270 | 0.7 | 529 | 0.7 | 3,092 | 0.5 |
| Gonorrhea | 36 | 0.8 | 210 | 0.5 | 460 | 0.6 | 3,531 | 0.6 |
| Chlamydia | <5 | 0.1 | 113 | 0.3 | 117 | 0.1 | 10,437 | 1.8 |
| Syphilis | 98 | 2.2 | 293 | 0.7 | 952 | 1.2 | 2,507 | 0.4 |
| Material Deprivation |  |  |  |  |  |  |  |  |
| Q1 | 877 | 20.4 | 4,941 | 12.5 | 34,944 | 44.4 | 112,058 | 19.7 |
| Q2 | 551 | 12.8 | 6,366 | 16.2 | 10,987 | 14 | 114,290 | 20.1 |
| Q3 | 436 | 10.2 | 7,305 | 18.6 | 8,948 | 11.4 | 120,207 | 21.1 |
| Q4 | 777 | 18.1 | 9,253 | 23.5 | 10,396 | 13.2 | 120,062 | 21.1 |
| Q5 | 1,651 | 38.5 | 11,507 | 29.2 | 13,455 | 17.1 | 102,236 | 18 |
| Social Deprivation |  |  |  |  |  |  |  |  |
| Q1 | 248 | 5.8 | 4,341 | 11 | 5,663 | 7.2 | 113,722 | 20 |
| Q2 | 304 | 7.1 | 5,250 | 13.3 | 7,254 | 9.2 | 109,803 | 19.3 |
| Q3 | 435 | 10.1 | 6,821 | 17.3 | 8,393 | 10.7 | 107,462 | 18.9 |
| Q4 | 692 | 16.1 | 8,424 | 21.4 | 16,709 | 21.2 | 115,068 | 20.2 |
| Q5 | 2,613 | 60.9 | 14,536 | 36.9 | 40,711 | 51.7 | 122,798 | 21.6 |
